# Modelling the impact of antimicrobial use and external introductions on commensal *E. coli* colistin resistance in small‐scale chicken farms of the Mekong delta of Vietnam

**DOI:** 10.1111/tbed.14558

**Published:** 2022-05-05

**Authors:** Jonathan Bastard, Nguyen Thi Nhung, Vo Be Hien, Bach Tuan Kiet, Laura Temime, Lulla Opatowski, Juan Carrique‐Mas, Marc Choisy

**Affiliations:** ^1^ Université Paris‐Saclay, UVSQ, Univ. Paris‐Sud, Inserm, CESP, Anti‐Infective Evasion and Pharmacoepidemiology Team Montigny‐le‐Bretonneux France; ^2^ Institut Pasteur Epidemiology and Modelling of Antibiotic Evasion Unit Paris France; ^3^ MESuRS laboratory Conservatoire national des arts et métiers Paris France; ^4^ PACRI unit Institut Pasteur Conservatoire national des arts et métiers Paris France; ^5^ Oxford University Clinical Research Unit Ho Chi Minh Vietnam; ^6^ Sub‐Department of Animal Health and Production, Dong Thap Vietnam; ^7^ Centre for Tropical Medicine and Global Health Oxford University Oxford UK

**Keywords:** antimicrobial resistance, colistin, *Escherichia coli*, poultry, Southeast Asia, spatial

## Abstract

Colistin is a critically important antimicrobial for human medicine, and colistin‐resistant *Escherichia coli* are commonly found in poultry and poultry products in Southeast Asia. Here, we aim at disentangling the within‐farm and outside‐farm drivers of colistin resistance in small‐scale chicken farms of the Mekong delta of Vietnam. Nineteen Vietnamese chicken farms were followed up along a whole production cycle, during which weekly antimicrobial use data were recorded. At the beginning, middle and end of each production cycle, commensal *E. coli* samples from birds were collected, pooled and tested for colistin resistance. Twelve models were fitted to the data using an expectation–maximization algorithm and compared. We further tested the spatial clustering of the occurrence of resistance importations from external sources using the local Moran's I statistic. In the best model, colistin resistance in *E. coli* from chickens was found to be mostly affected by importations of resistance, and, to a lesser extent, by the use of antimicrobials in the last 1.73 weeks [0.00; 2.90], but not by the use of antimicrobials in day‐olds, nor their colistin resistance carriage from hatchery. The occurrence of external source importations proved to be sometimes spatially clustered, suggesting a role of local environmental sources of colistin resistance.

## 1 INTRODUCTION

Antimicrobial resistance (AMR) is a major threat worldwide and it is hastened by excessive antimicrobial use (AMU) (Ferri et al., 2017). Antimicrobials intended for veterinary use amount to a substantial fraction of total AMU (Carrique‐Mas et al., 2020; ECDC et al., 2017), and AMU and AMR in food‐producing animals are thought to contribute to the burden of resistance in human populations (Puyvelde et al., 2017), especially in low‐ and middle‐income countries (Nadimpalli et al., 2018).

Polymyxins, a class of antibiotics that includes colistin (polymyxin E), are last resort antimicrobials for multidrug‐resistant Gram‐negative bacterial infections, and are thus classified as critically important antimicrobials for human medicine by the World Health Organization (WHO, 2019). The fact that resistance of Enterobacterales to colistin is reported in livestock worldwide, specifically in Asia, is a serious public health concern (Apostolakos & Piccirillo, 2018; Boeckel et al., 2019; Kempf et al., 2016).

In particular, high levels of colistin resistance have been reported in *Escherichia coli* collected from chicken products (Yamaguchi et al., 2018) and chicken flocks (Kawahara et al., 2019; Nguyen et al., 2016) in Southeast Asia. Chicken is the most consumed meat in this region and is the livestock production sector that grows the fastest (OECD & FAO, 2020). Moreover, the zoonotic potential of colistin‐resistant bacteria carried by poultry has been shown in chicken farms in Vietnam (Trung et al., 2017).

Previous studies have investigated the factors that drive AMR in food‐producing animals and, among them, some have specifically focused on colistin resistance in chicken farms. Hypothesized factors include AMU (Majewski et al., 2020; Nguyen et al., 2016), carry‐over from a previous flock raised in the same building (Mo et al., 2019) or colistin resistance in day‐old chicks on arrival at the farm (Baron et al., 2014). A major limit of these studies is that they rarely consider external sources of contamination that may impact levels of resistance measured on farms, for example, transmission from the surrounding environment such as water sources, or from humans to the flock (Lee et al., 2020). Indeed, such accidental resistance importations from outside the farm are difficult to detect directly (as compared with measuring AMU for instance), requiring the use of specific methods to account for these unobserved data. The multiplicity of potential resistance selection and transmission mechanisms renders the identification of an association between use and resistance difficult to unravel at the scale of a farm, and spatial (in addition to temporal) analyses become necessary (Rosenkrantz et al., 2019; Singer et al., 2006).

In this study, we analyze longitudinal AMU and colistin resistance data collected from small‐scale commercial chicken farms of the Mekong delta in Vietnam, a region that accounts for ∼13% of Vietnam total chicken production (Truong et al., 2021). We apply an expectation–maximization algorithm to (i) assess the contributions of within‐flock factors (such as AMU) on colistin resistance in chickens during production and (ii) estimate for each farm the probability of importation of resistance from external sources. We finally assess the spatial distribution of this probability, to investigate the presence of local hotspots of introduction of colistin resistant bacteria into chicken flocks.

## 2 MATERIALS AND METHODS

### 2.1 Study farms and data collection

**FIGURE 1:**
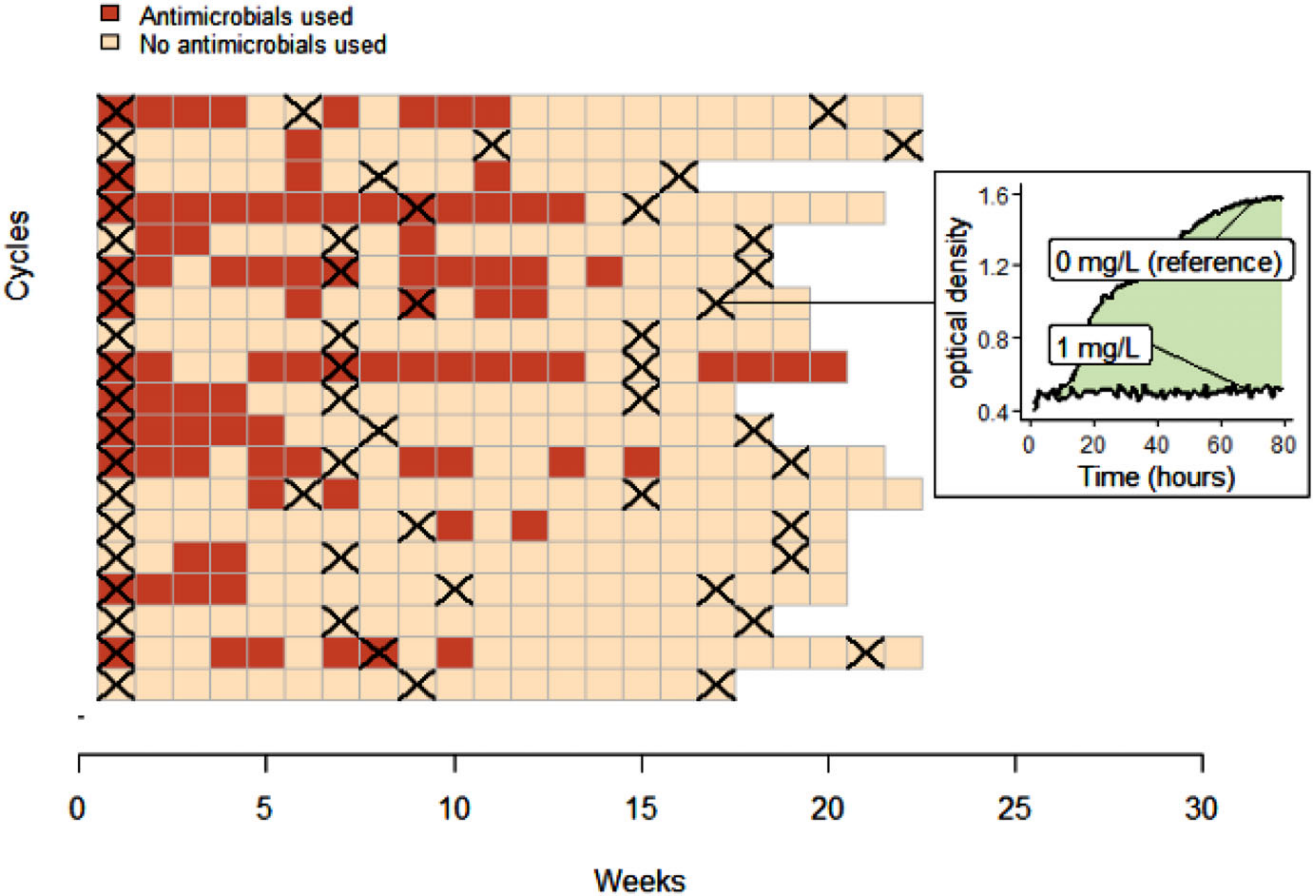
Description of the data collection. Rows correspond to the 19 chicken production cycles. The colour of the square indicates the weekly use of antimicrobials. Black crosses indicate weeks of pooled faecal sample collection, which happened on three occasions for each cycle: on restocking, in the middle and at the end of the production cycle. For each pooled sample, growth curves of *E. coli* were determined by optical density, in presence of different colistin concentrations. The metric of colistin resistance in our study is the area between the growth curve under 1 mg/L of colistin and the growth curve under 0 mg/L of colistin (see Section 2.2)

The study took place in 19 farms raising chickens for meat located in the districts of Thap Muoi and Cao Lanh, Dong Thap province (Mekong Delta region of Vietnam), as part of the baseline phase of the ViParc research project described in Carrique‐Mas and Rushton (2017) and Phu et al. (2021). Briefly, all farmers registered in the two districts and raising more than 100 meat chickens as single age (i.e. 200–300 farm owners in each district) were invited to participate to the project. 102 farms were included in the ViParc project (Phu et al., 2021), among which 19 were randomly selected for this study. These 19 farms correspond to small‐scale commercial productions, with flock size between 100 and 2000 chickens of the same age (all‐in/all‐out system), raised over production cycles of 4–5 months in a dedicated area of the farm, and fed and watered manually. The use of antimicrobials in the flocks was recorded on a weekly basis by the farmers (Figure 1). All commercial containers were kept to ensure the generation of reliable AMU data (Cuong et al., 2019).

In each flock, three rounds of faeces sampling were performed: on arrival of day‐old chicks (restocking), in the middle and at the end of the production cycle (Figure 1). The day before each sampling visit, farmers were asked to place a tarp on the pen's floor, in order to collect commensal *E. coli* from chickens’ gut flora. Indeed, *E. coli* is a classical indicator of AMR in commensal gut bacteria in human and animal populations (EFSA & ECDC, 2021; Tadesse et al., 2012). The faeces samples collected from the whole flock on the tarp were pooled for each sampling round of each flock, resulting in 19 × 3 samples.

### 2.2 Estimation of colistin resistance in samples

From each pooled sample of faeces, 30–50 colonies of *E. coli* were selected and pooled to have a resulting sample representative of the population of commensal *E. coli* in the collected faeces on each farm at each time point. Growth curves of *E. coli* were determined by growing a standardized suspension and measuring its OD_600nm_ (optical density), in presence of different colistin concentrations: 0, 0.5, 1, 2, 4, 8, 12 and 16 mg/L (Supplementary Material SM1). For each sample, this measure was repeated twice and averaged. The method, validated by Nhung et al. (2021), aims to obtain an aggregated estimate of colistin resistance among a representative sample of the whole *E. coli* population circulating in the guts of all the chickens of the flock of each farm and at each time point.

The chosen metric of colistin resistance is the area between the growth curve of *E. coli* in presence of colistin and the reference growth curve in absence of colistin (Figure 1 and SM1). If, for a given non‐null colistin concentration, the growth curve is lower than the reference, the bacteria are considered susceptible to this concentration of colistin.

Because the objective is to explain the variance in the colistin resistance measured in samples, we first pre‐select concentrations showing both low‐growth (susceptible bacteria) and high‐growth curves (resistant bacteria) (SM1). In order to increase the power of our analysis, we then select the colistin concentration *c* that provides the maximal standard deviation of our resistance metric. A robustness analysis on the value of *c* is also performed (see Section 2.7).

Because the distribution of the metric is bimodal (see SM1), we use a Gaussian Mixture Model with two components to determine the probability for each sample to belong to the ‘resistant’ or ‘susceptible’ categories. The value of probability 0.5 is the threshold for considering a sample either as ‘resistant’ or ‘susceptible’ (SM1).

Note that the classification we make between susceptible and resistant strains is based on a statistical rationale only (based on the bimodality of the resistance metric) and not on a clinical breakpoint. Indeed, the objectives of this study are to assess the factors increasing or decreasing the level of colistin resistance, independently of the threshold conventionally used to characterize colistin resistant versus susceptible bacteria.

### 2.3 Models

For each sample (*i*,*j*) collected in the flock *i* at the *j*th sampling round of the production cycle (*j*∈{2;3}), that is, 2 × 19 = 38 samples, we aim at explaining the observed resistance status *R_ij_*, 0 for ‘susceptible’ and 1 for ‘resistant’.

The resistance status is assumed to follow the Bernoulli trial:

$$R_{\mathrm{ij}}∼B\left(p_{\mathrm{ij}}\right)$$

where *p_ij_*, the probability for bacteria in sample (*i*,*j*) to be resistant, is defined as:

$$\;p_{ij}=\frac{1}{1+e^{-[\mu +\alpha .L_{ij}+\varphi .S_{i}+\sum_{k\in \left\{1;\;j-1\right\}}\left(\lambda_{k}.R_{ik}\right)]}}\;\;\mathrm{if}\;\;Z_{ij}=0$$

$$\;p_{ij}=\eta \;\mathrm{if}\;\;Z_{ij}=1$$

where (Table 1):

- *α* is the effect of using antimicrobials before the sampling week. *L_ij_* is the number of weeks of AMU during the *β* weeks preceding the *j*th sampling round in flock *i* (see SM2 for illustration), where *β* is estimated (Table 1). *L_ij_* is defined as:
$$\;L_{ij}=\int_{t_{ij}-1-\beta }^{t_{ij}-1}U_{i}\left(t\right)dt\;$$

where *t_ij_* is the week of sampling round *j* in flock *i*. *U_i_* (*t*) = 1 if antimicrobials were used in the flock *i* during week *t*, and *U_i_* (*t*) = 0 otherwise (SM2).

- *φ* is the effect of using antimicrobials at the beginning of the production cycle. *S_i_* is a measure of the AMU occurring during the first *δ* weeks of the production cycle of flock *i* (SM2), where *δ* is estimated (Table 1). *S_i_* is similarly defined as:
$$\;S_{i}=\int_{0}^{\delta }U_{i}\left(t\right)dt\;$$
- *λ*_1_ and *λ*_2_ represent the effect of the resistance in the previous samples of the same flock (assuming potential autocorrelation), *R_i_*_1_ and *R_i_*_2_ (Table 1).
- *μ* is the baseline within‐flock acquisition of colistin resistance, without AMU nor previous resistance measured (Table 1).
- *Z* is a latent (unobserved) binary variable, such that *Z_ij_*_ _= 1 (*j*∈{2;3}) if an unobserved event of importation from external sources increased the load of colistin resistance in the flock *i* between the sampling rounds *j* − 1 and *j*, and *Z_ij_*_ _= 0 otherwise. Therefore, if *Z_ij_*_ _= 1, the probability of the sample to be resistant is *η*, a fixed parameter with a value close to 1 (but cannot be equal to 1 to allow model fitting) (Table 1). On the contrary, if no importation event occurred (*Z_ij_*_ _= 0), the probability of resistance *p_ij_* follows a logistic function accounting for AMU and the resistance in the previous samples of the same flock.

**TABLE 1:** Parameters used in the analysis

Parameter	Description	Value	Unit	Domain of definition
*c*	Colistin concentration chosen for the colistin resistance metric in samples	1 (See Section 2.2 and SM1)	mg/L	–
*α*	Effect of AMU shortly before sampling (recent AMU) on colistin resistance	Estimated	–	]−∞; +∞[
*β*	Number of weeks considered for the effect of recent AMU (see SM2)	Estimated	Weeks	]0; 5[
*φ*	Effect of AMU during the first few weeks of the production cycle (initial AMU) on colistin resistance	Estimated	–	]−∞; +∞[
*δ*	Number of initial weeks in the production cycle considered for the effect of initial AMU (see SM2)	Estimated	Weeks	]0; 5[
*η*	Probability of colistin resistance of the sample after an importation of resistance from external sources	0.999 (assumption)	–	–
*λ*_k_	Effect of the colistin resistance previously measured in the *k*th round of sampling (autocorrelation)	Estimated	–	]−∞; +∞[
*μ*	Baseline within‐flock acquisition of colistin resistance, without AMU nor previous resistance measured	Estimated	–	]−∞; +∞[
*D*	Distance used to compute the local' Moran I statistic (see Section 2.6)	Tuned	km	[0.1; 24]

We consider 12 versions of the model, described in Table 2, that are particular cases of the full model detailed above. We note *θ* the set of model parameters.

**TABLE 2:** Variants of the model

Model	Previous resistance		Antimicrobial use	
	*R_i_*_1_	*R_i_*_2_	Initial	Recent
1				
2	X			
3	X	X		
4			X	
5	X		X	
6	X	X	X	
7				X
8	X			X
9	X	X		X
10			X	X
11	X		X	X
12	X	X	X	X

All models are particular cases of the full model (model 12) described in Section 2. Crosses indicate, for each model in rows, the inclusion in the model of the variables in columns. The different models are all possible combinations of the variables, excepted those based on *R_i_*_2_ without *R_i_*_1_. Indeed, those would represent a situation where the autocorrelation of *E. coli* resistance in chickens would affect sampling round 3 but not sampling round 2.

### 2.4 Estimation using the expectation–maximization algorithm

For a given model, let *Y*_m_ be the vector of values of the observed variables for sample *m* = (*i*,*j*), including *R_m_* and the explanatory variables. Because our model includes both observed *Y* and unobserved data *Z*, we use an expectation–maximization algorithm (Dempster et al., 1977) to (i) estimate the parameters of the model, noted *θ*, and (ii) determine for each sample m the probability to have *Z_m_*_ _= 1, that is, the probability that an external source importation of resistance occurred. This algorithm comprises two steps detailed in SM3. In the expectation step, we determine for each observation the probability that an external source importation occurred given a set *θ* ^(^ *^s^* ^)^ of parameters for the model, that is, *P*(*Z*_m _= 1|*Y_m_*, *θ* ^(^ *^s^* ^)^). In the maximization step, we estimate *θ* ^(^ *^s^* ^+1)^ by maximizing the complete‐data LogLikelihood (also called *Q* function, see SM3), based on the value of *P*(*Z_m_*_ _= 1|*Y_m_*, *θ* ^(^ *^s^* ^)^) computed in the previous expectation step. The algorithm is terminated once the complete‐data likelihood of the model has converged.

### 2.5 Model selection

Classic criteria for models’ comparison and selection, such as the Akaike information criterion (AIC), most often depend on the likelihood based on the observed data, and are not directly suited for incomplete data problems like ours. Therefore, we use the IC_H,Q_ criterion, defined in Ibrahim et al. (2008), that is similar to the AIC if we set the penalty term to two times the effective number of parameters (see SM4 for details). The model presenting the lowest value of IC_H,Q_ is selected as the best model.

To validate the method, we simulate mock resistance data according to four scenarios, and apply the E–M algorithm and model selection process to each of these scenarios. The method is validated if, for each scenario, the best model retrieves the simulated relationships between explanatory variables and the outcome (see details in SM5).

### 2.6 Investigation of local spatial clusters of the occurrence of external source importations of resistance

As a result of the E–M algorithm, for each observation (sample) *m* = (*i*,*j*), we obtain an estimate of *P* ^inf^ *_m_* = *P*(*Z_m_*_ _= 1|*Y_m_*, θ*), the probability of occurrence of an external source importation at convergence of the E–M algorithm, in the best model. Here, we test if the values of *P* ^inf^ from each location are spatially clustered. We compute the local Moran's I statistic (Anselin, 1995; Zhu et al., 2019), defined for any observation *m* as:

$$\;I_{m}=\frac{\left(P_{m}^{\inf}-\bar{P^{\inf}}\right)}{S^{2}}\;\;\sum_{n}W_{mn}\left(P_{n}^{\inf}-\bar{P^{\inf}}\right)$$

where *S* ^2^ is the variance of *P* ^inf^, and *W_mn_* = 1 (resp. = 0) if the distance between samples *m* and *n* is ≤*D* (resp. > *D*). *D* is a tuning parameter that is searched exhaustively (Table 1).

The more positive (resp. negative) *I_m_* is, the more *P* ^inf^ *_m_* is similar (resp. dissimilar) to neighbouring values of *P* ^inf^. This allows to classify observations as high–high, low–low, high–low, low–high or not significant. A high–high (resp. low–low) point has a high (resp. low) value of *P* ^inf^ surrounded by other high (resp. low) values of *P* ^inf^, which corresponds to a significant spatial cluster of high (resp. low) probability of external source importation. A high–low (resp. low–high) point has a high (resp. low) value of *P* ^inf^ surrounded by low (resp. high) values of *P* ^inf^, forming an outlier. To compute this statistic, we use the R package *spdep* version 1.1‐3 (Bivand & Wong, 2018).

### 2.7 Robustness of the results

We test the robustness of our results with regard to the assumed value of two parameters: the colistin concentration used to measure colistin resistance, *c* (among the pre‐selected values, see Section 2.2), and the assumed probability of colistin resistance of the sample after an external source importation, *η*. We run the analysis five additional independent times using respectively (*c; η*) = (2; 0.999), (4; 0.999), (1; 0.99), (2; 0.99) and (4; 0.99), on top of the baseline analysis using (*c; η*) = (1; 0.999) (see Table 1). All analyses are performed using R version 3.6.1 (R Core Team, 2019).

## 3 RESULTS

### 3.1 Measure of colistin resistance

Among the seven non‐null colistin concentrations used to determine *E. coli* growth curves from the pooled sampled, we pre‐select concentrations 1, 2 and 4 mg/L because they are the only ones showing both low‐growth (susceptible bacteria) and high‐growth curves (resistant bacteria) (SM1).

The distributions of the resistance metric for each concentration of colistin are shown in SM1. The standard deviation is 18.1 for 1 mg/L, 17.4 for 2 mg/L and 15.0 for 4 mg/L. We therefore select the resistance metric under *c* = 1 mg/L as measure of resistance in all samples.

As a result, out of 57 (19 × 3) samples, 31 (54.4%) are classified as resistant and 26 (45.6%) as susceptible. Among the 38 (19 × 2) samples belonging to the 2nd and 3rd sampling rounds and included in the model (see Section 2.3), 21 are classified as resistant and 17 as susceptible.

### 3.2 Model selection and fit

Our validation study shows that our methodology allows to successfully identify the scenario generating the simulated data (see SM5). We can therefore apply the method to the real field data.

**FIGURE 2:**
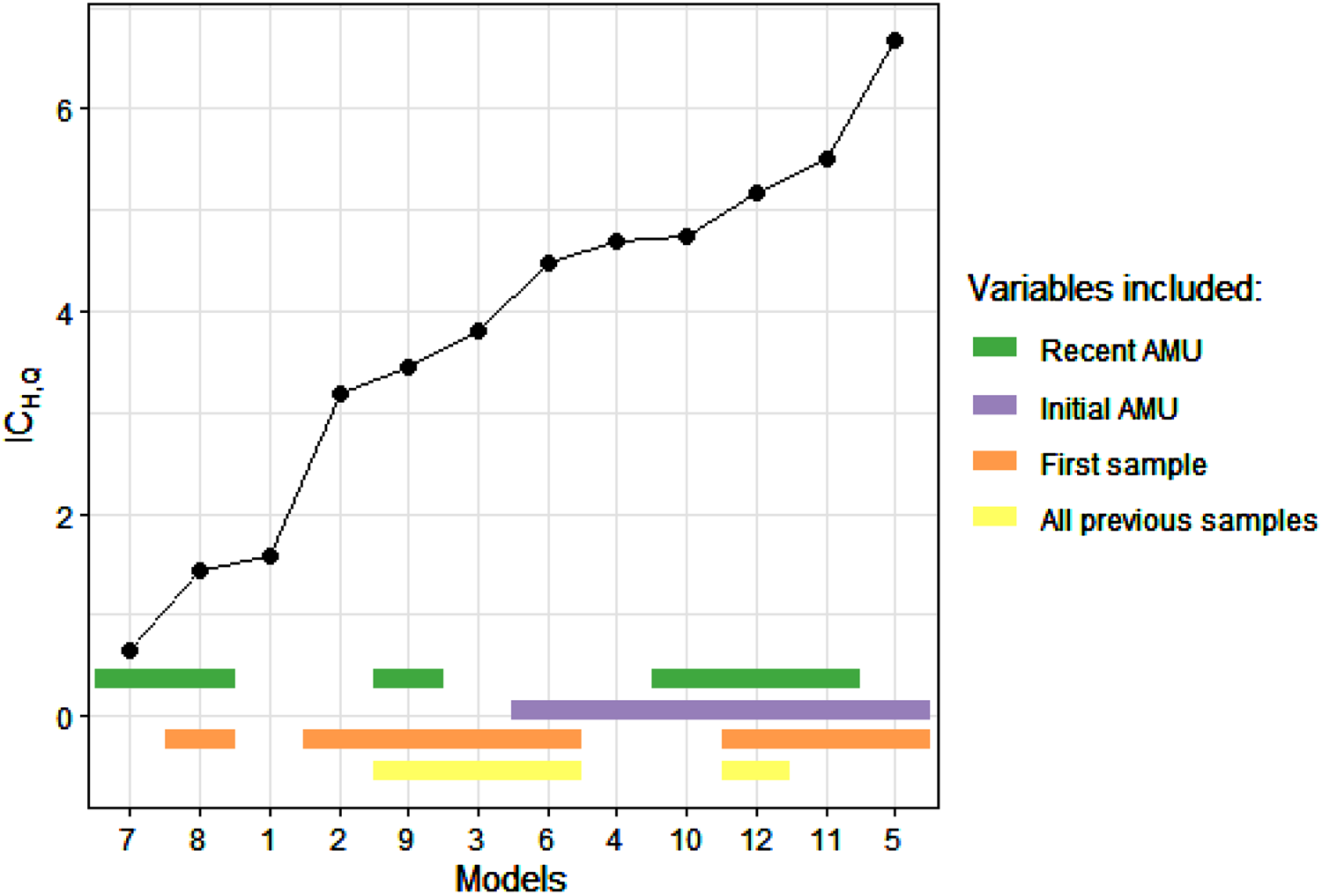
Ranking of the models. Models are ranked by increasing IC_H,Q_ order. The model with the lowest IC_H,Q_ value (Model 7) is considered to be the best model. The variables included are displayed for each model

The model presenting the lowest IC_H,Q_ is Model 7 (Figure 2 and SM6). In this model, parameters estimates are: $\hat{\mu }$ = −12.1 (95% confidence interval: [−33.3; −4.05]), $\hat{\alpha }$ = 9.78 [4.61; +∞[ and $\hat{\beta }$ = 1.73 [2.80 × 10^−7^; 2.90]. Therefore, the use of antimicrobials during the 1.73 weeks preceding a sampling round is positively associated with colistin resistance in the sample.

The recent use of antimicrobials is a variable included in the two models with the lowest IC_H,Q_. The models including an effect of initial AMU always have a higher value of IC_H,Q_ than their counterpart without this effect (Figure 2 and SM6).

### 3.3 Local spatial clusters of the probability of external source importation

**FIGURE 3:**
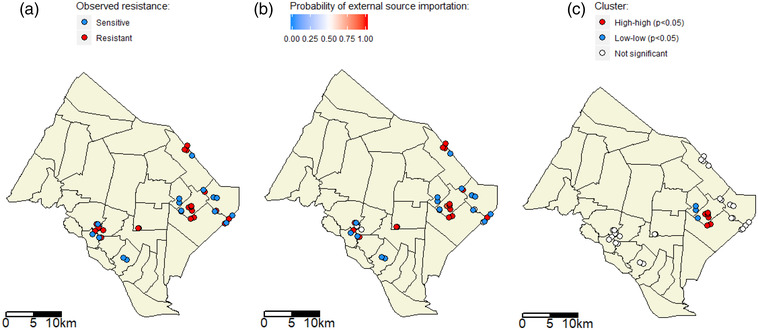
Geographical distribution of colistin resistance, and of the probability of external source importation of resistance into farms. For the farms studied, located in the districts of Thap Muoi and Cao Lanh (Vietnam): susceptibility to colistin measured in samples (panel a), probability that an external source importation occurred in the flock (panel b) and spatial clusters of this probability (panel c). In panel c, high–high (resp. low–low) dots correspond to a significant spatial cluster of high (resp. low) probability of external source importation. For visualization purposes, overlapping dots are randomly shifted by up to 670 m from their actual coordinates

For the computation of the local Moran's I statistic, the tuning distance parameter *D* is set to 2.3 km. Figure 3 presents the spatial distribution of the colistin resistance metric (Figure 3(a)), and of the probability of external source importation *P* ^inf^ estimated in Model 7 for each observation (sample) with the E–M algorithm (Figure 3(b)). Six (resp. four) data points show significant spatial high–high (resp. low–low) clustering (*p* < .05) (Figure 3(c)). This indicates that there is one local spatial cluster of high probability of external source importation, and one local spatial cluster of low probability. For all other observations, *P* ^inf^ is not significantly spatially clustered.

### 3.4 Robustness of the results

Results are similar to the baseline analysis for all five tested values of (*c; η*) (see details in SM7). Indeed, in all cases, the selected model – that is, the model with the lowest IC_H,Q_ – is also Model 7. Estimates of parameters are similar, especially $\hat{\beta }$ that ranges from 1.63 to 1.69. It means the estimated antimicrobials effect, including its duration, does not depend on the assumption for (*c; η*). Moreover, we always detect a high–high cluster of the probability of external source importation *P* ^inf^ in the same geographical area than in the baseline analysis. However, low–low spatial clustering is not always retrieved as in the baseline analysis (SM7).

## 4 DISCUSSION

In this study, we explore the contributions of within‐farm and outside‐farm factors to the phenotypic colistin resistance observed in commensal *E. coli* isolated from chicken farms of the Mekong delta of Vietnam. Our methodology was validated using a simulation study (SM5), allowing to have confidence in our results.

### 4.1 Main findings

Some studies (Nguyen et al., 2016; Poolperm et al., 2020) have suggested an association between AMU and colistin resistance of Enterobacterales isolated from food‐producing animals in Southeast Asia. Here, the collection of detailed and reliable AMU data on a weekly basis allows us to account for the dynamics of the effect of AMU on AMR. We find that our measure of colistin resistance is associated with the use of antimicrobials during the 1.73 weeks preceding the sample collection, similarly to (Nhung et al., 2021). The reason may be that AMU selects for colistin resistant *E. coli* strains that were already present at low level or that were introduced to the farm. This suggests that mitigating the global exposure of chickens to antimicrobials – and not only the exposure to colistin – during the production cycle would help preserve the effectiveness of colistin. The effect of other antibiotic classes on colistin resistance could be explained by mechanisms of co‐selection.

However, AMU in the first weeks is not found to lastingly affect colistin resistance measured later in the production cycle. This could be due to the non‐persistence of the effect of AMU on colistin resistance over time, after the interruption of antimicrobials use, as was described before (Poolperm et al., 2020). Consistently, our estimate of parameter *β* (1.73 weeks) suggests that the effect of AMU does not last longer than 2 weeks in the chicken flocks. This might be explained by the evolutionary costs associated with resistance in bacteria (Melnyk et al., 2015). Nonetheless, these findings must be confirmed by other studies exploring the impact of AMU at different periods of the production cycle.

A second result is that, during the course of our study, most of the colistin resistance observed in chickens (17 out of 21 of resistant samples) was actually not explained by within‐farm AMU, but imported from outside the flock. Although we cannot determine the nature of these events of infection, an explanation may be the introduction of colistin‐resistant bacteria to the flock from other animals or humans, as it is known that antibiotic‐resistant bacteria can be transmitted between species (Kawahara et al., 2019; Woolhouse et al., 2015).

The source of these unexplained external source importations may also be the environment, for example as a result of the contamination of water sources or the soil with antibiotic residues or resistance genes (Liang et al., 2020; Sun et al., 2017; Zhou et al., 2019). In our study, the presence of a local spatial cluster of high probabilities of external source importations was suggested from the computation of the local Moran's I statistic (Figure 3). It supports the hypothesis of a common environmental source of colistin resistance in this geographical area. A previous publication (Rosenkrantz et al., 2019) also found spatial drivers of AMR in food‐producing animals, whereas another did not (Huber et al., 2021). More studies should be carried out to investigate the relative contribution of geographical factors to AMR, as compared with within‐farm factors, for instance using genomic data to detect introductions of new *E. coli* clones to the flock.

The contribution of antimicrobials administered at the hatchery level to AMR in poultry production farms has been suggested (Baron et al., 2014; Okorafor et al., 2019; Seo et al., 2020; Verrette et al., 2019). However, in our analysis, the best model does not include an effect of colistin resistance carriage in day‐old chicks on its carriage later in the production cycle. This does not necessarily mean that the phenomenon does not occur, but simply that in our farms it is not the predominant mechanism.

Because we use a statistical classification to distinguish susceptible from resistant strains, and not a clinical breakpoint, the percentage of resistant samples presented here should not be interpreted as a prevalence result comparable to other prevalence surveys. This said, our method has the advantage to overcome the usual difficulties of measuring susceptibility to colistin due its poor diffusion in agar (Apostolakos & Piccirillo, 2018; Kempf et al., 2016). Furthermore, our results are not affected by the exact value chosen for the parameter *c* used to perform the statistical classification (see Section 3.4).

### 4.2 Limitations

A limitation of our work is that we use only phenotypic data. Genomic data would be useful to test the hypothesis of a common environmental source of colistin resistance in different farms for instance. Such an approach is not possible with the design used, as samples are pooled. Conversely, this pooling method has the advantage to provide a representative aggregate of the colistin resistance observed in the gut flora of all chickens of the flock.

Another limitation of our model is that it does not account for the antimicrobials used in the previous production cycles of each farm, as this information was not systematically collected. In the future, it would be interesting to build model variants accounting for such data, as a previous study (Mo et al., 2019) suggested that AMR in successive broiler flocks of a same farm could be associated.

### 4.3 Implications for AMR study and control

Our study confirms the importance of considering the landscape scale when investigating the determinants of AMR (Singer et al., 2006; Zhao et al., 2021). Here, we could interpret our results in terms of sources and sinks of resistance. Farms in which colistin resistance can be explained by within‐farm factors (such as AMU) might act as sources of colistin resistance to their environment and therefore to other farms. Inversely, farms where the resistance is only driven by events of external source importation may act as sinks of colistin resistance, exposed to environmental contamination with resistance genes. Geographical areas with high environmental exposure can be characterized as local hotspots of colistin resistance.

The detection of such local hotspots of resistance suggests that it would be more efficient to account for geographical factors in AMR mitigation interventions. Indeed, if a farm is located in a geographical hotspot of AMR, the benefits of mitigation strategies implemented in the farm (e.g., AMU reduction) might be cancelled by the influence of its neighbours (Rosenkrantz et al., 2019). Therefore, it would be interesting to compare the efficacy of interventions (e.g., raising AMR awareness or veterinary advice) implemented in a large percentage of farms of a small area, *versus* in a smaller percentage of farms of a larger area.

## CONFLICT OF INTEREST

The authors declare no conflict of interest.

## ETHICS STATEMENT

The authors confirm that the ethical policies of the journal, as noted on the journal's author guidelines page, have been adhered to. The project was granted ethics approval by the Oxford Tropical Research Ethics Committee (OXTREC) (Ref. 5121/16).

## Supporting information

Supporting information

## Data Availability

The data that support the findings of this study are available from the corresponding author upon reasonable request.
