# Supplementary material for: Modelling the impact of antimicrobial use and external introductions on commensal E. coli colistin resistance in small‐scale chicken farms of the Mekong delta of Vietnam
Source: Transbound Emerg Dis. 2022 May 5;69(5):e2185–94. doi: 10.1111/tbed.14558 (PMC9790599; doi:10.1111/tbed.14558)
Supplement: Supplementary file 1 — Supporting information [file TBED-69-e2185-s001.pdf]

Supplementary material

Modelling the impact of antimicrobial use and external introductions on commensal *E. coli* colistin resistance in small-scale chicken farms of the Mekong delta of Vietnam

Jonathan Bastard, Nguyen Thi Nhung, Vo Be Hien, Bach Tuan Kiet, Laura Temime, Lulla Opatowski, Juan Carrique-Mas, Marc Choisy

SM1. Colistin resistance in samples

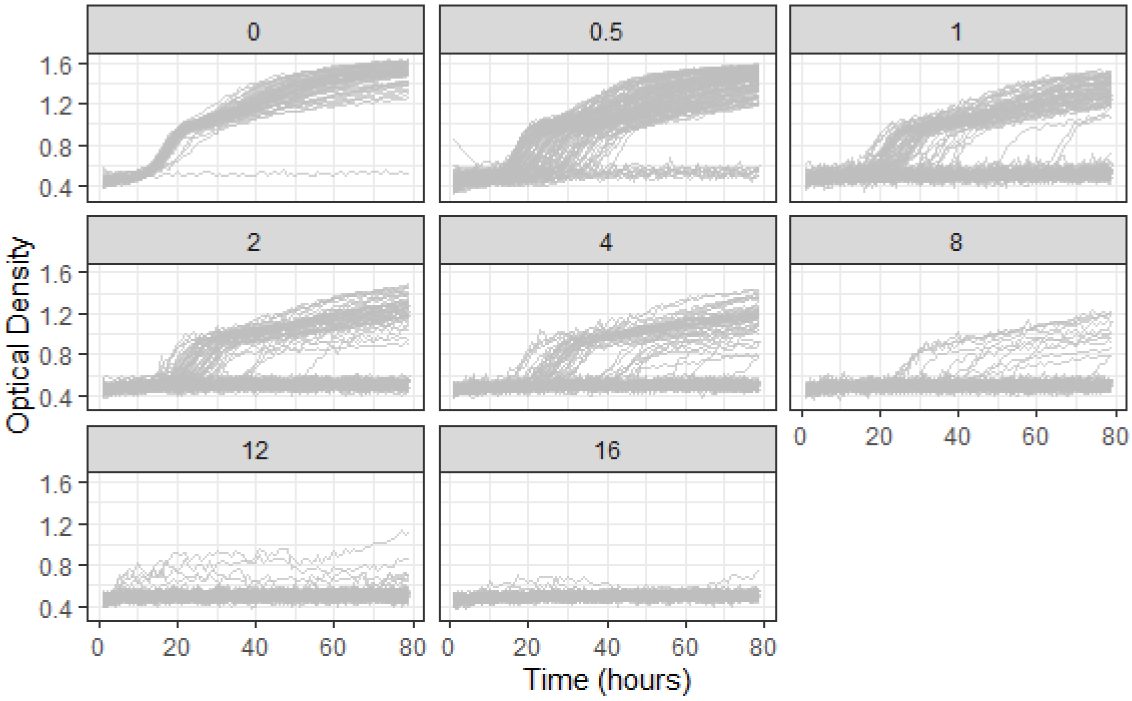

Figure S1. Optical Density curves for each colistin concentration (in mg/L). *Each curve (i.e. each sample for each concentration) was replicated twice. Only concentrations 1, 2 and 4 mg/L show a significant amount of both high-growth (*E. coli *are resistant at this concentration) and low-growth (*E. coli *are susceptible at this concentration) curves. Concentration 0 is the reference.*

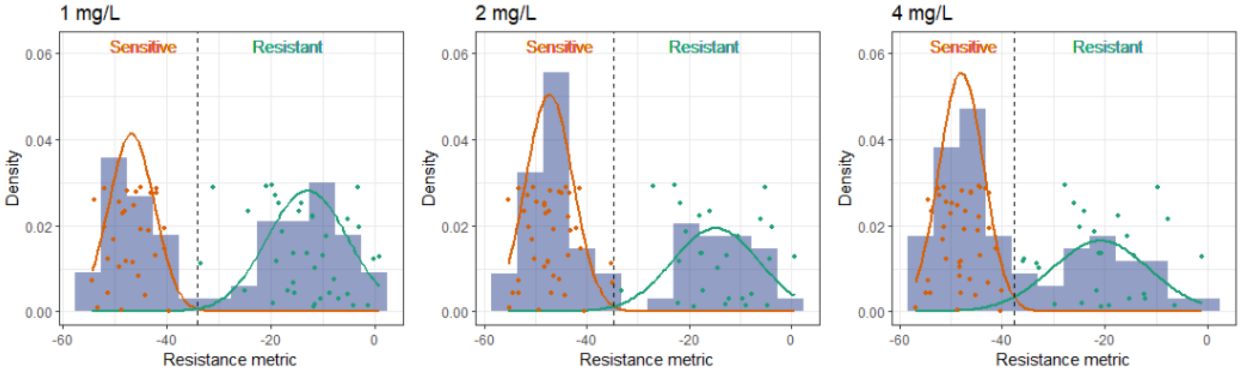

Figure S2. Distribution of resistance metric values for each colistin concentration. *For each concentration, the resistance metric is the area between the growth curve under this concentration of colistin, and the growth curve in absence of colistin (reference). Only the distributions for 1, 2 and 4 mg/L are displayed, as they are the only concentrations presenting similar amounts of high-growth and low-growth curves (see Figure S1). The y position of dots is random and just for displaying purposes. For each concentration, we use a two-component Gaussian Mixture Model (GMM) to discriminate samples between two categories: Susceptible and Resistant. The corresponding fit curves are displayed in colors. For each concentration, the dashed line is the threshold delimiting susceptible (left) from resistant (right) samples. It is the value for which the probability to belong to each component of the GMM is 0.5. The colistin concentration value we select for the rest of the analysis is* c = *1 mg/L, as it leads to a standard deviation in the resistance metric values of 18.1, compared to 17.4 and 15.0 for concentrations 2 mg/L and 4 mg/L respectively. A robustness analysis on the value of c is also performed (see section 2.7 of the main document).*

SM2. Effect of antimicrobial use in the model

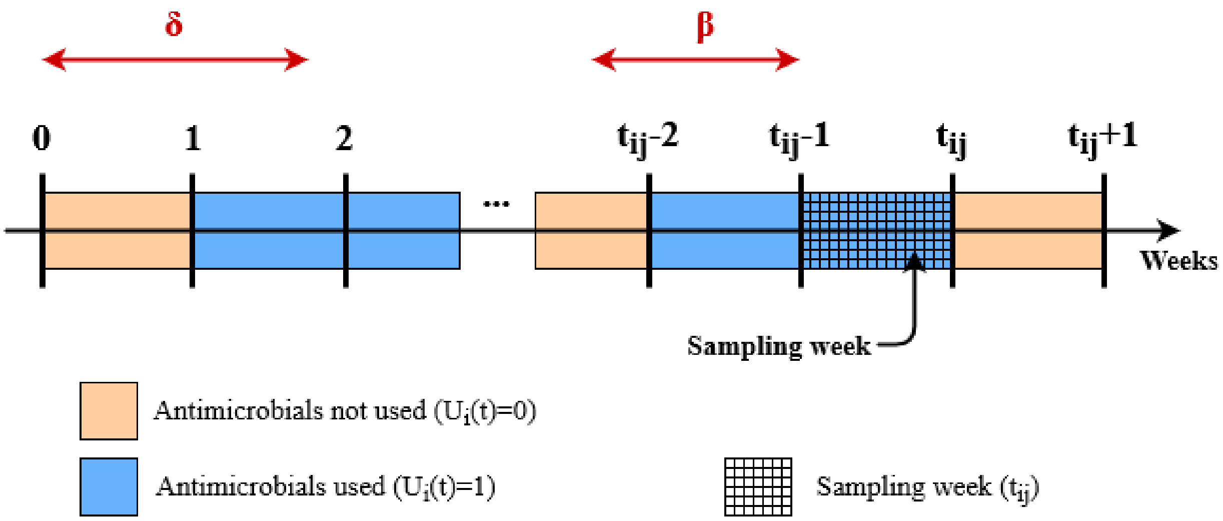

Figure S3. Two components of the effect of AMU on colistin resistance in the model*: the AMU in the β weeks before the sampling week t_{ij} (L_{ij}), and the AMU during the first δ weeks of the production cycle (S_i).*

SM3. Details on the Expectation-Maximization algorithm

The algorithm is depicted in Figure S4. For a given model, Y is the observed variables, and Y_m their value for sample m=(i,j), including R_m and the explanatory variables detailed in section 2.3 (main document). Z is a latent binary variable such that $Z_m = 1$ if an importation of resistance from external sources occurred for sample m, and $Z_m = 0$ otherwise. θ is the set of parameters for the model.

At any step s of the E-M algorithm, the complete-data LogLikelihood, named Q function, is defined as (Dempster et al., 1977):

$$Q(\theta, \theta^{(s)}) = E[ln\, P(Y, Z|\theta) \quad |Y, \theta^{(s)}]$$

where $E[\bullet \; | Y, \theta^{(s)}]$ is the conditional expectation with respect to $P(Z \; | Y, \theta^{(s)})$, i.e.:

$$\begin{aligned} Q(\theta, \theta^{(s)}) &= \sum_m E[ln\, P(Y_m, Z_m|\theta) \quad |Y_m, \theta^{(s)}] \\ &= \sum_m [P(Z_m = 0|Y_m, \theta^{(s)}).\, ln\, P(Z_m = 0, Y_m|\theta) \; + \; P(Z_m = 1|Y_m, \theta^{(s)}).\, ln\, P(Z_m = 1, Y_m|\theta)] \end{aligned}$$

The E-M algorithm is composed of two steps repeated in loop until convergence (Figure S4). The algorithm is started with $P(Z_m = 1 \; | Y_m, \theta^{(0)}) = 0.01$, but the exact value chosen here does not affect the convergence (tests were done with values ranging from 0.01 to 0.99). Then, the following steps are repeated until convergence:

- **Maximization step:** because the value of $P(Z_m = 1 \; | Y_m, \theta^{(s)})$ is known from the previous E-step (or from initialization if s = 0), it is possible to find the set of parameters $\theta^{(s+1)}$ maximizing the complete-data LogLikelihood $Q(\theta, \theta^{(s)})$:

$$\theta^{(s+1)} = \underset{\theta}{argmax}(Q(\theta, \theta^{(s)}))$$

- **Expectation step:** The probability of resistance importation from external source, i.e. $P(Z_m = 1 \; | Y_m, \theta^{(s+1)})$ (the distribution of the missing data), is computed for each sample using the set of parameters $\theta^{(s+1)}$ estimated from the previous Maximization step:

$$\begin{aligned} P(Z_m = 1|Y_m, \theta^{(s+1)}) &= \frac{P(Z_m = 1, Y_m|\theta^{(s+1)})}{P(Y_m|\theta^{(s+1)})} \\ &= \frac{P(Z_m = 1, Y_m|\theta^{(s+1)})}{P(Z_m = 0, Y_m|\theta^{(s+1)}) \; + \; P(Z_m = 1, Y_m|\theta^{(s+1)})} \\ &= \frac{\eta^{R_m}.\,(1-\eta)^{1-R_m}}{M_{m,s+1}^{R_m}.\,(1-M_{m,s+1})^{1-R_m} \; + \; \eta^{R_m}.\,(1-\eta)^{1-R_m}} \end{aligned}$$

where η is the probability of resistance when an external source importation occurs (see Table 1, main document), $M_{m,s+1}$ the probability of resistance predicted by the logistic function (with parameters $\theta^{(s+1)}$) for sample m when no external source importation occurs (see section 2.3, main document), and R_m is the resistance observed (0 or 1) for sample m.

And:

$$P(Z_m = 0|Y_m, \theta^{(s+1)}) = 1 - P(Z_m = 1|Y_m, \theta^{(s+1)})$$

Steps E and M are repeated until the complete-data LogLikelihood Q converges. The convergence is defined as having five times in a row the difference between the complete-data LogLikelihood values of steps s+1 and s less than 0.01.

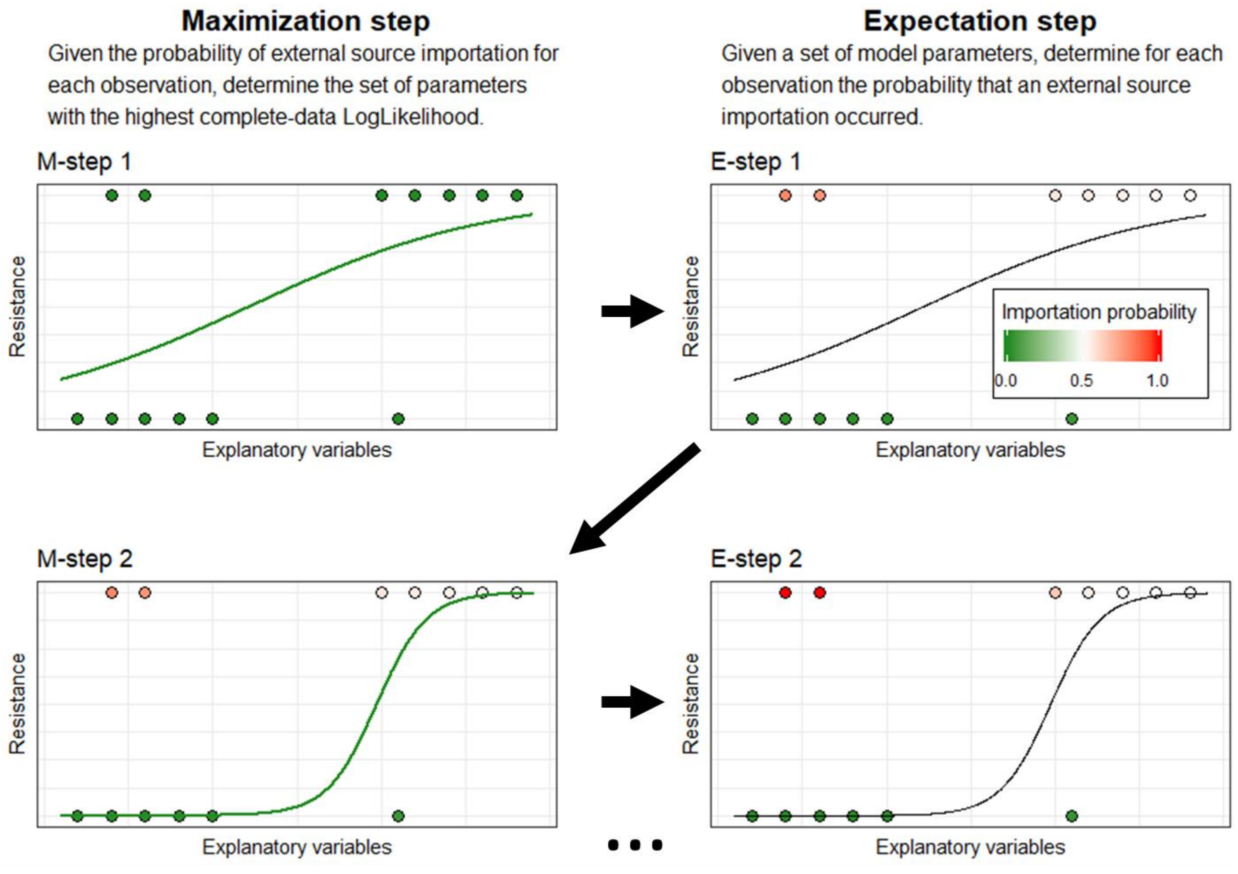

Figure S4. Description of the Expectation-Maximization algorithm. *In the M-step, the complete-data LogLikelihood (i.e. the Q function), is maximized given the current value of the probability of external source importation of resistance. In the E-step, the probability of external source importation (i.e. the distribution of the missing data) is determined given the current estimates of the parameters. The M-step and E-step are alternated until convergence of the complete-data LogLikelihood.*

SM4. Model selection in a missing data situation

Once the convergence of the E-M algorithm has been reached for all models, we aim at comparing the models. The Akaike Information Criterion (AIC) is based on the observed data likelihood. Here, as there is a latent variable Z, we maximize the complete-data (and not observed data) LogLikelihood at each Maximization step. Instead, we use the $IC_{H,Q}$ criterion defined in (Ibrahim et al., 2008).

First, it has to be noted that, for any step s of the algorithm:

$$\begin{aligned}
Q(\theta,\theta^{(s)}) &= \sum_m [P(Z_m=0|Y_m,\theta^{(s)}).\,ln\,P(Z_m=0,Y_m|\theta) \;+\; P(Z_m=1|Y_m,\theta^{(s)}).\,ln\,P(Z_m=1,Y_m|\theta)]\\
&= \sum_m [P(Z_m=0|Y_m,\theta^{(s)}).\,ln\,[P(Z_m=0|Y_m,\theta).\,P(Y_m|\theta)] \;+\; P(Z_m=1|Y_m,\theta^{(s)}).\,ln\,[P(Z_m=1|Y_m,\theta).\,P(Y_m|\theta)]]\\
&= \sum_m [[ln\,P(Y_m|\theta)].\,[P(Z_m=0|Y_m,\theta^{(s)})+P(Z_m=1|Y_m,\theta^{(s)})]\\
&\quad + P(Z_m=0|Y_m,\theta^{(s)}).\,ln\,P(Z_m=0|Y_m,\theta) \;+\; P(Z_m=1|Y_m,\theta^{(s)}).\,ln\,P(Z_m=1|Y_m,\theta)]\\
&= \sum_m [ln\,P(Y_m|\theta)] \;+\; H(\theta,\theta^{(s)})\\
&= L_{obs}(\theta) \;+\; H(\theta,\theta^{(s)})
\end{aligned}$$

where $L_{obs}(\theta)$ is the LogLikelihood of the observed data and:

$$H(\theta,\theta^{(s)}) = \sum_m [P(Z_m=0|Y_m,\theta^{(s)}).\,ln\,P(Z_m=0|Y_m,\theta) \;+\; P(Z_m=1|Y_m,\theta^{(s)}).\,ln\,P(Z_m=1|Y_m,\theta)]$$

Therefore, when convergence was reached:

$$Q(\theta^*,\theta^*) = L_{obs}(\theta^*) \;+\; H(\theta^*,\theta^*)$$

where θ* is the converged set of parameters.

(Ibrahim et al., 2008) define the $IC_{H,Q}$ as:

$$IC_{H,Q} = -2Q(\theta^*,\theta^*) + 2H(\theta^*,\theta^*) + C^*(\theta^*)$$

where C*(θ*) is the model penalty term that we set to two times the number of parameters. Therefore, we retrieve the AIC.

Q(θ*,θ*) is directly available from the Maximization step at convergence. In section 2.6 (main document), we defined for each sample m: P^{inf}_m = P(Z_m=1 | Y_m, θ*), then:

$$H(\theta^*,\theta^*) = \sum_m [P_m^{inf}.\,ln(P_m^{inf}) + (1-P_m^{inf}).\,ln(1-P_m^{inf})]$$

SM5. Method validation

To validate the method, we simulate mock resistance data according to four scenarios, and apply the E-M algorithm and model selection process to each of these scenarios. The method is validated if, for each scenario, the best model retrieves the simulated relationships between explanatory variables and the outcome.

- Scenario 1:

In the first scenario, we simulate random resistance data, representing a situation with no relationship between the explanatory variables and the resistance. In this scenario, for each (i,j), $R_{ij} \backsim B(0.5)$.

After application of the E-M algorithm and comparison of models, the best model selected (with the lowest $IC_{H,Q}$) is Model 1, i.e. the null model, as expected (Figure S5).

- Scenario 2:

In the second scenario, we simulate resistance data such that, using model 7, recent AMU predicts the resistance with a 85% positive predictive value and a 85% negative predictive value. In details, let:

$$f_1(\theta_1, i, j) = \frac{1}{1+\exp(-(\mu_1+\alpha_1.L_{ij}))}$$

where $\theta_1 = (\mu_1, \alpha_1, \beta_1)$ is fixed.

For each (i,j), $R_{ij} \sim B(p_{1ij})$, where $p_{1ij} = 0.85$ if $f_1(\theta_1, i, j) \geq 0.5$ and $p_{1ij} = 0.15$ if $f_1(\theta_1, i, j) < 0.5$.

When R_{ij} is simulated as such, and our method is applied on the mock data, the best model selected in this scenario is Model 7 (Figure S5). Therefore, the simulated relationship between the explanatory variables and the outcome is retrieved with our method.

- Scenario 3:

In the third scenario, we simulate resistance data such that, using model 4, initial AMU predicts the resistance in later samples of the same flock, with a 85% positive predictive value and a 85% negative predictive value. In details, let:

$$f_2(\theta_2, i, j) = \frac{1}{1+\exp(-(\mu_2+\varphi_2.S_i))}$$

where $\theta_2 = (\mu_2, \varphi_2, \partial_2)$ is fixed.

For each (i,j), $R_{ij} \sim B(p_{2ij})$, where $p_{2ij} = 0.85$ if $f_2(\theta_2, i, j) \geq 0.5$ and $p_{2ij} = 0.15$ if $f_2(\theta_2, i, j) < 0.5$.

When R_{ij} is simulated as such, and our method is applied on the mock data, the best model selected in this scenario is Model 4 (Figure S5). Therefore, the simulated relationship between the explanatory variables and the outcome is retrieved with our method.

- Scenario 4:

In the fourth scenario, we simulate resistance data such that, using model 2, the carriage of colistin resistance in day-old chicks (arriving from hatchery) predicts the resistance with a 85% positive predictive value and a 85% negative predictive value. In details, let:

$$f_3(\theta_3, i, j) = \frac{1}{1+\exp(-(\mu_3+\lambda_3.R_{i1}))}$$

where $\theta_3 = (\mu_3, \lambda_3)$ is fixed.

For each (i,j), $R_{ij} \sim B(p_{3ij})$, where $p_{3ij} = 0.85$ if $f_3(\theta_3, i, j) \geq 0.5$ and $p_{3ij} = 0.15$ if $f_3(\theta_3, i, j) < 0.5$.

When R_{ij} is simulated as such, and our method is applied on the mock data, the best model selected in this scenario is Model 2 (Figure S5). Therefore, the simulated relationship between the explanatory variables and the outcome is retrieved with our method.

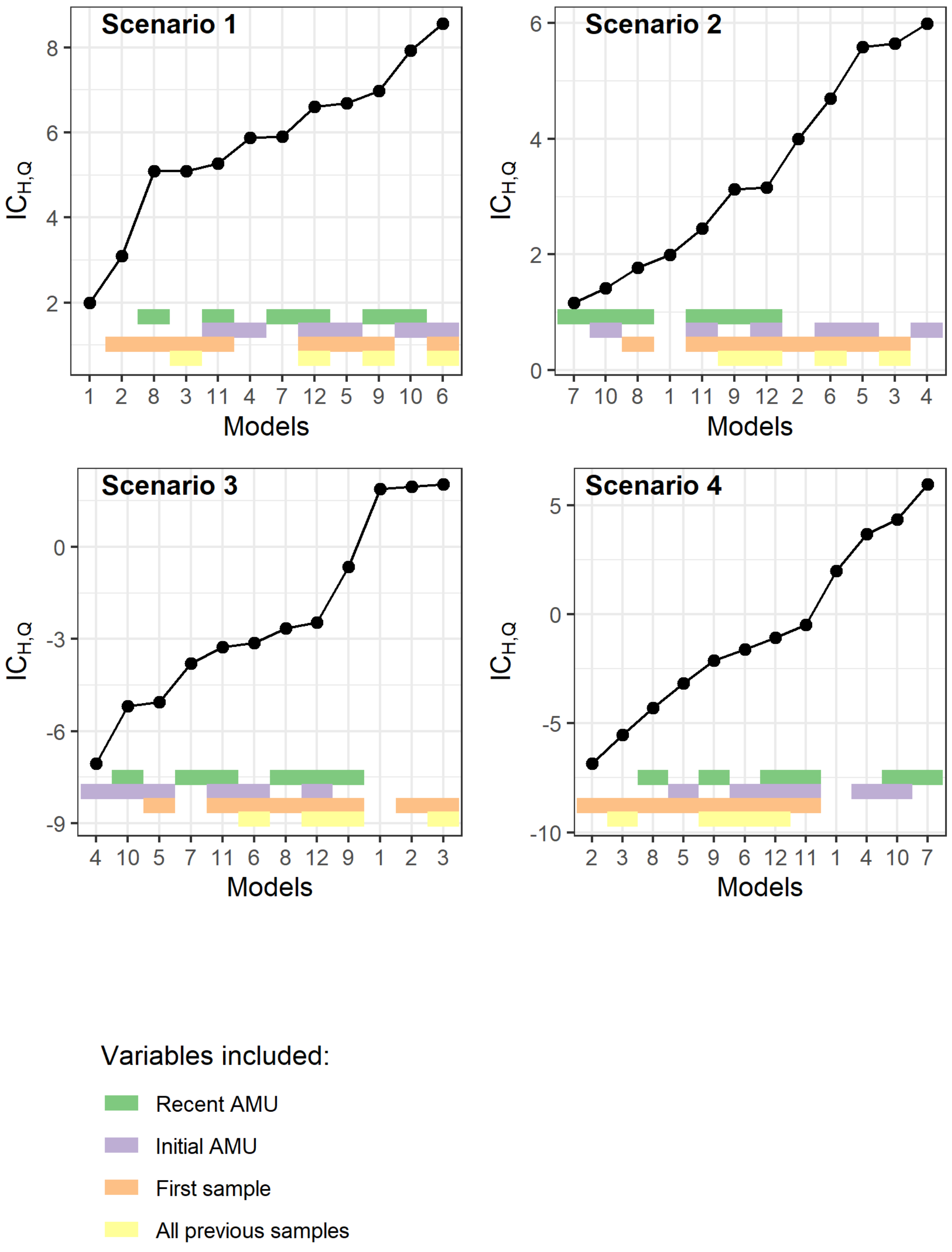

Figure S5. Ranking of the models in method validation scenarios. *For each scenario, models, fitted to mock data, are ranked by increasing $IC_{H,Q}$ order. For each scenario, the model with the lowest $IC_{H,Q}$ value (respectively Model 1, 7, 4 and 2 in scenario 1, 2, 3 and 4) is considered to be the best model. The variables included are displayed for each model.*

SM6. Additional results of model selection

Table S1 presents the values of the complete-data LogLikelihood and of the $IC_{H,Q}$ criterion in the baseline analysis. Model 7 has the lowest $IC_{H,Q}$ value and is therefore selected as the best model.

Table S1. Model selection. *Complete-data LogLikelihood* (i.e. *Q function) and $IC_{H,Q}$ criterion for each model.*

**Model**	**Complete-data LogLikelihood**	**$IC_{H,Q}$**
**1**	-6.60	1.58
**2**	-5.68	3.20
**3**	-7.26	3.81
**4**	-4.04	4.68
**5**	-4.17	6.68
**6**	-2.33	4.47
**7**	-0.69	0.66
**8**	-0.45	1.44
**9**	-0.45	3.44
**10**	-1.18	4.74
**11**	-0.97	5.51
**12**	-0.77	5.17

SM7. Robustness of the results

Using c = 2 mg/L (resp. c = 4 mg/L), 10 (resp. 9) samples are classified as susceptible whereas they are classified as resistant in the baseline analysis with c = 1 mg/L, including 3 (resp. 2) belonging to the first round of sampling, 4 (resp. 4) to the second and 3 (resp. 3) to the third.

Therefore, for a given value of η, the results of the E-M algorithm are the same with c = 2 mg/L and c = 4 mg/L in models 1, 4, 7 and 10 only, i.e. the models that do not include the variable R_{i1} (see Table 2, main document). As a consequence, in Model 7, for any given value of η, the parameter estimates and the local spatial clusters detected are the same with c = 2 mg/L and c = 4 mg/L.

The results of the model selection for the five tested values of (c; η) are presented in Table S2. Model 7 shows the lowest $IC_{H,Q}$ value in all cases and is therefore selected as the best model. Table S3 presents the points estimates of Model 7 parameters for all tested values of (c; η). Finally, Figure S6 shows the local spatial clustering of the probability of external source importation of resistance, to compare with Figure 3C of the main document.

Table S2. Model selection for different values of (c; η). *The $IC_{H,Q}$ value is given for each model and each value of* (c; η)*. The model with the lowest $IC_{H,Q}$ value is considered to be the best model. Whatever the value of* (c; η)*, model 7 is always the best model.*

**Model**	**$IC_{H,Q}$**				
	***η* = 0.999**		***η* = 0.99**		
	***c* = 2 mg/L**	***c* = 4 mg/L**	***c* = 1 mg/L**	***c* = 2 mg/L**	***c* = 4 mg/L**
**1**	2.00	2.00	1.58	1.95	1.95
**2**	3.99	3.99	3.20	3.93	3.94
**3**	4.98	5.91	3.79	4.92	5.84
**4**	6.00	6.00	4.82	5.95	5.95
**5**	7.98	7.99	6.80	7.90	7.94
**6**	4.78	9.90	5.45	5.84	9.83
**7**	**0.59**	**0.59**	**1.43**	**1.13**	**1.13**
**8**	1.30	1.31	2.81	5.25	2.28
**9**	0.64	3.31	5.86	2.18	7.24
**10**	3.16	3.16	5.89	3.64	3.64
**11**	5.30	5.16	6.80	5.61	5.63
**12**	4.49	7.16	8.92	5.43	7.61

***Table S3. Values of $\hat{\mu}$, $\hat{\alpha}$ and $\hat{\beta}$ estimated in model 7 for different values of* (c; η).** *In model 7, for any given value of* η*, the parameter estimates are the same with* c = *2 mg/L and* c = *4 mg/L (see SM7 text).*

		Point estimates of parameters		
		$\hat{\mu}$	$\hat{\alpha}$	$\hat{\beta}$
**η = 0.999**	**c = 2 mg/L**	-14.0	11.5	1.69
	**c = 4 mg/L**	-14.0	11.5	1.69
**η = 0.99**	**c = 1 mg/L**	-6.30	5.41	1.67
	**c = 2 mg/L**	-8.12	6.93	1.63
	**c = 4 mg/L**	-8.12	6.93	1.63

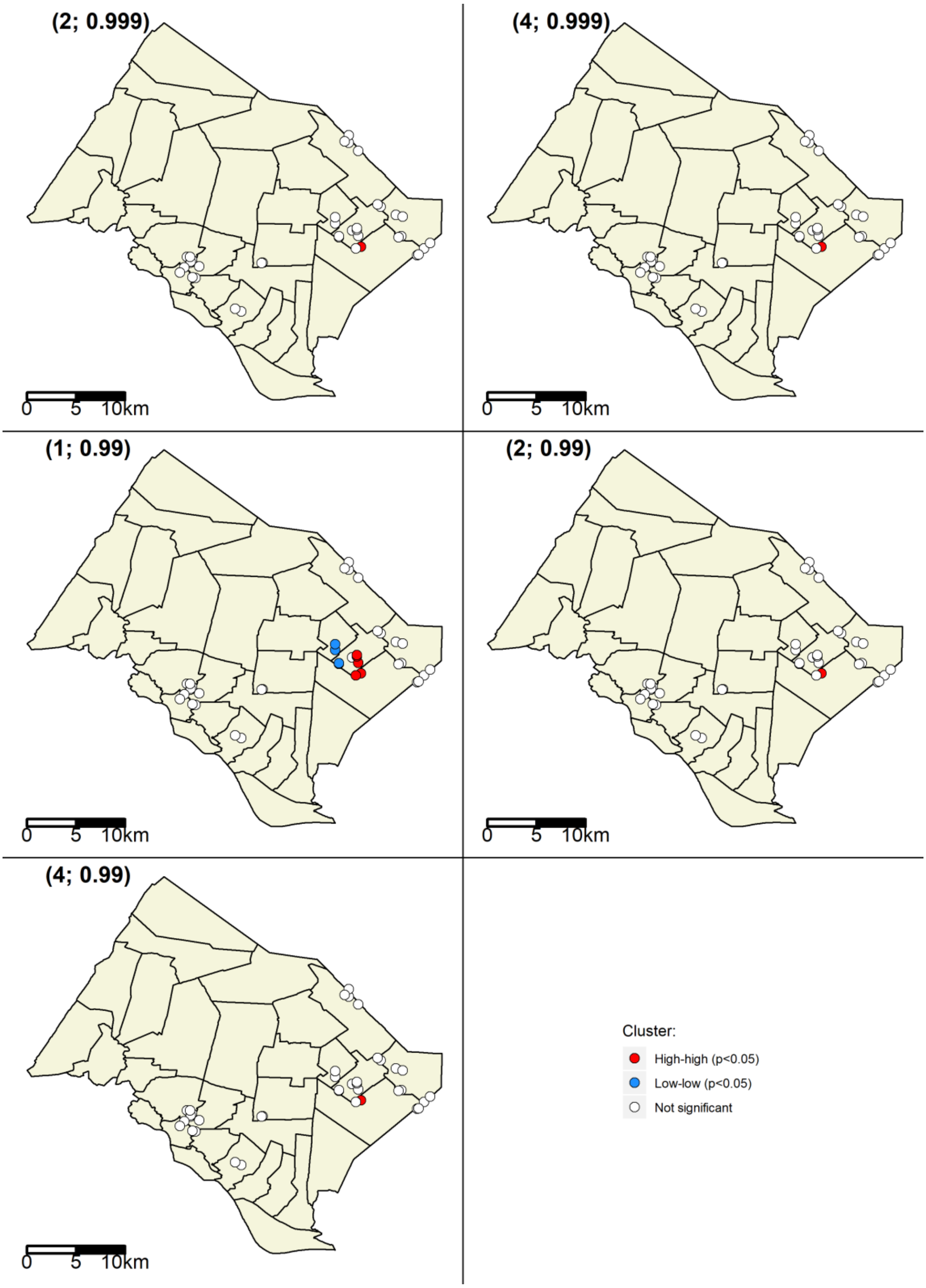

***Figure S6. Local spatial clustering of the probability of external source importation of resistance for different values of* (c; η).** *Local spatial clustering can be either high-high (red), low-low (blue) or not significant. One significant (colored) dot is enough to identify a cluster. For visualization purposes, overlapping dots are randomly shifted by up to 670 meters from their actual coordinates. Compared to the baseline analysis (Figure 3, main document), we always detect a high-high cluster of the probability of importation, in the same geographical area. However, low-low spatial clustering is not always retrieved as in the baseline analysis.*

References of the Supplementary Material

Dempster, A. P., Laird, N. M., & Rubin, D. B. (1977). Maximum Likelihood from Incomplete Data via the EM Algorithm. *Journal of the Royal Statistical Society. Series B (Methodological)*, *39*(1), 1–38. JSTOR.

Ibrahim, J. G., Zhu, H., & Tang, N. (2008). Model Selection Criteria for Missing-Data Problems Using the EM Algorithm. *Journal of the American Statistical Association*, *103*(484), 1648–1658. PubMed. https://doi.org/10.1198/016214508000001057
